# Exploring the “Tip of the Tongue” and “Feeling of Knowing” Phenomena During Advanced Aging: The Interplay of Age of Acquisition, Vocabulary and Verbal Fluency

**DOI:** 10.3390/bs15121686

**Published:** 2025-12-05

**Authors:** Carlos Rojas, Yasna Sandoval, Bárbara Farías, Gabriel Lagos, Álvaro Poza, Bernardo Riffo, Ernesto Guerra

**Affiliations:** 1 Department of Health Rehabilitation Sciences, University of Bío-Bío, Chillán 3780000, Chile; crojas@ubiobio.cl; 2 Department of Speech Therapy, University of Valparaíso, Valparaíso 2340000, Chile; alvaro.poza@uv.cl; 3 Department of Spanish, University of Concepción, Concepción 4030000, Chile; bernardo@udec.cl; 4 Center for Advanced Research in Education (CIAE), University of Chile, Santiago 8320000, Chile; ernesto.guerra@ciae.uchile.cl

**Keywords:** tip of the tongue, feeling of knowing, age of acquisition, vocabulary, verbal fluency, older adults

## Abstract

Background/Objectives: The “tip of the tongue” (TOT) and “feeling of knowing” (FOK) phenomena were cognitive experiences that notably affected word retrieval, particularly among older adults. The study aimed to investigate the influences of age of acquisition (AoA), vocabulary size, and verbal fluency on the frequency and nature of TOT and FOK occurrences as individuals aged. Methods: A behavioral experiment was conducted based on the two-step word retrieval framework established by Gollan and Brown in 2006. Early and late acquisition words were utilized to induce tip-of-the-tongue phenomena and the feeling of knowing. Additionally, vocabulary and verbal fluency tests were administered. Sixty monolingual older adults participated in the study (35 female, 25 male; mean age: 77.66 years). Mixed-effects linear regressions had been used to analyze the data. Results: The logistic regression analysis identified age of acquisition as the most significant predictor of TOT and FOK experiences (*p* < 0.0001), highlighting that earlier vocabulary acquisition enhanced retrieval efficiency. Notable interactions between vocabulary size and verbal fluency illustrated that increased lexical knowledge diminished reliance on age of acquisition for successful retrieval. Conclusions: The findings underscore the importance of early vocabulary acquisition as a protective factor against cognitive decline in older adults, emphasizing the necessity for interventions aimed at enhancing vocabulary and fluency. This study contributed valuable insights into the cognitive mechanisms underlying language retrieval and suggested that fostering rich linguistic environments throughout life could facilitate better cognitive health in aging populations.

## 1. Introduction

The ‘tip of the tongue’ (TOT) phenomenon is a common cognitive experience involving an individual’s temporary inability to recall a familiar word or name (Abrams & Davis, 2016; Brown, 2012; Ouyang et al., 2020). It often leads to frustration as people are aware of the information but cannot access it at that moment (Barry et al., 2025). Research suggests that people experiencing TOT utilize these moments to infer the characteristics of the elusive information, effectively treating them as cognitive heuristics to guide their retrieval efforts (Kim & Choi, 2021). Common theories surrounding TOT posit that such states arise from the attributions individuals make based on the partial information they can access. For example, when someone retrieves some attributes of a word but not the whole word, they often overestimate their knowledge of it, believing that retrieval is imminent (Rousseau & Kashur, 2021).

In addition, the “feeling of knowing” (FOK) is a cognitive phenomenon that relates to the subjective belief that one is unable to retrieve a piece of information yet possesses sufficient associative connections to recognize that information when it is presented (Hart, 1965; Koriat, 2000; Pournaghdali et al., 2025). FOK is closely related to metacognition, as it reflects an individual’s awareness of their own cognitive state. This phenomenon is considered an integral part of memory processes, serving as a subjective indicator of potentially successful retrieval. When an individual experiences a FOK, they may have a strong sense that the elusive information is temporarily inaccessible, often leading to strategies aimed at triggering retrieval, such as the activation of related concepts or cues (Schwartz, 2010). In contrast, the TOT phenomenon is characterized by the frustrating experience of knowing that a specific word or name is known yet being unable to access it at that moment (Cleary et al., 2021; Ryals et al., 2021). 

The distinction between a total omission (TOT) and a false omission (FOK) is noteworthy, particularly with regard to their emotional and cognitive implications (Schwartz & Cleary, 2016; Schwartz, 2010). TOTs not only embody the frustration of failing to retrieve a known word, but also the accompanying sensations of urgency and expectation of imminent retrieval (Koriat, 2000). In contrast, FOK experiences do not generally involve the same immediate frustration; rather, they encourage a reflective approach to the associations that lead to successful retrieval. These differences emphasize that, although both phenomena result from retrieval attempts, they activate different cognitive processes. Neuroimaging studies reveal that different brain regions are engaged during each state (Koriat, 2000; Schwartz, 2010; Schwartz & Cleary, 2016). The cognitive underpinnings of these two experiences suggest that TOTs highlight precise shortcomings in lexical retrieval, whereas FOKs indicate broader metacognitive evaluations of potential future retrieval success.

From an aging perspective, TOTs are recognized as being more prevalent in older adults and are tied to various psycholinguistic factors, such as age of acquisition and vocabulary size, which influence retrieval efficiency (Rahman et al., 2023; Shafto et al., 2007). The increased frequency of TOTs among older individuals may also reflect declines in functional brain processes related to retrieval monitoring and cognitive control (Rahman et al., 2023). Conversely, FOKs can manifest in different contexts, demonstrating varying strategies depending on an individual’s overall cognitive health. In summary, although TOTs and FOKs are both types of retrieval failure, their distinct characteristics warrant further exploration and clarification. TOTs emphasize immediate retrieval challenges with strong emotional influences (imminent feeling of producing the word + frustration), whereas FOKs offer insights into expected recognition potential and cognitive strategies for future retrieval attempts (metacognition + reflection).

Empirical research into both phenomena would suggest that psycholinguistic factors such as age of acquisition (AoA; Wang et al., 2024), vocabulary size (Rojas et al., 2023a), and verbal fluency (Frankenberg et al., 2021) might play critical roles in determining whether an individual might experience a TOT or an FOK. Specifically, the research implies that an earlier AoA is positively correlated with enhanced fluency and retrieval effectiveness, which might indicate that individuals with a richer early vocabulary experience fewer retrieval failures (Rojas et al., 2023b). This relationship could highlight the significance of both TOT and FOK within the broader context of cognitive aging, suggesting that older adults may be more likely to experience an increased frequency of these phenomena. However, the precise roles of AoA, vocabulary size, and verbal fluency in influencing lexical retrieval in older age would require more thorough exploration.

### 1.1. TOTs, FOKs and Age-Related Changes in Cognitive Functions

TOT and FOK states may be particularly prevalent among older adults, with empirical evidence suggesting that the frequency of these episodes increases with age (Pournaghdali et al., 2025; Schwartz, 2010; Schwartz & Cleary, 2016). These occurrences raise concerns for individuals as they may indicate potential cognitive decline (Abrams & Davis, 2016; Kim & Choi, 2021). Understanding the mechanisms that underlie TOT and FOK experiences is essential for comprehending how aging affects language retrieval processes (Ouyang et al., 2020).

Various cognitive theories have been proposed to explain the mechanisms underlying TOT and FOK experiences in the context of aging. A prominent explanation suggests that these states may arise from partial activation of the target word, where TOT states develop from the activation of phonological features related to the target word while complete retrieval remains out of reach (Medeiros, 2024). This partial activation can evoke feelings of familiarity and intensify sensations of being on the verge of recollection, despite the inability to access the full target. Another aspect to consider involves the retrieval of fragments of the target word, such as initial letters or syllables, which serve as mnemonic cues, potentially heightening the feeling of impending recollection (Claxton, 2023; Cleary & Claxton, 2015; Huebert et al., 2023). Such fragments underscore the complexity of lexical retrieval processes and illustrate the challenges associated with word recovery.

Moreover, challenges related to phonological processing may be implicated in the occurrence of TOT and FOK states, particularly among older individuals (Abrams & Davis, 2016; Kim & Choi, 2021). These difficulties may indicate that weakened connections between phonological representations and their lexical counterparts contribute to increased retrieval challenges as one ages (Rojas et al., 2023a, 2023b). This phonological decline highlights the complex interplay between lexical meaning and sound, underscoring the importance of phonetic accessibility in language processes. Additionally, TOT states may involve metacognitive components, where individuals experience heightened awareness of their cognitive state, leading to a sense of familiarity and a belief that retrieval is imminent (Schwartz, 2010).

As the global population continues to age, understanding how age-related effects manifest in cognitive functions becomes increasingly vital. Studies indicate that aging may disrupt essential brain regions involved in language processing, such as the left inferior frontal gyrus and posterior temporal areas critical for lexical retrieval and phonological access (Burke & Graham, 2012). Age-related declines in fluid intelligence—such as verbal fluency—processing speed, and executive function may exacerbate the challenges associated with language retrieval (Martin et al., 2021; Tucker-Drob et al., 2022). Therefore, it is crucial for researchers to investigate not only the frequency of TOT episodes but also the specific contexts and cognitive strategies that may influence how these experiences manifest (Salthouse, 2013).

Another pertinent consideration in the study of TOTs is the phenomenon of repeated occurrences for specific words. Some studies suggest an error-learning component, where individuals may unintentionally develop cognitive strategies—heuristics that reinforce retrieval failures for specific lexical items, particularly those that are rarely used or contextually unusual (Catling et al., 2021; Wang et al., 2024). This reinforcement may result in maladaptive retrieval strategies, where cognitive effort devoted to accessing a familiar word leads to increased frustration and decreased performance (Abrams & Davis, 2016; Kim & Choi, 2021).

Furthermore, the relationship between TOT and FOK experiences, age, and cognitive decline underscores the need for further investigation into how specific characteristics of lexical items—including age of acquisition (AoA), frequency of use, and semantic associations—interact with cognitive aging. Evidence suggests that words learned earlier in life might be more accessible and may exhibit greater resilience to age-related retrieval challenges (Catling et al., 2021; Wang et al., 2024). However, there remains insufficient evidence regarding how these dynamics play out in the later stages of old age.

### 1.2. Interactions Between AoA, Vocabulary and Verbal Fluency

The concept of AoA can be seen as a significant factor in understanding vocabulary dynamics and its influence on cognitive processing, particularly regarding the frequency of TOT or FOK experiences. Research might indicate that words learned early in life are generally associated with faster and more accurate lexical retrieval compared to those acquired later (Brysbaert et al., 2000; Elsherif et al., 2023; Johnston & Barry, 2006). These findings point to the protective role of early vocabulary acquisition, which might assist in building a more resilient cognitive foundation that potentially mitigates age-related cognitive decline (Elsherif et al., 2023). The complexities inherent in cognitive networks enable the early acquisition of words to be more effectively integrated, thereby enhancing retrieval efficiency and allowing older individuals to access their linguistic resources more competently (Verhagen & Van Stiphout, 2022).

The dynamics of vocabulary may also reveal that some lexical items are more likely to elicit TOT experiences than others. Specifically, it seems that proper names and low-frequency words might be particularly prone to these states, primarily due to their less frequent use in linguistic contexts (Gollan et al., 2011; Rojas et al., 2023b).

Moreover, the interaction between AoA and vocabulary size might illustrate how linguistic proficiency could moderate retrieval challenges. As individuals presumably expand their vocabularies, the negative implications of AoA on retrieval probabilities might diminish. This suggests that those with larger vocabularies may not depend as heavily on AoA to retrieve words, indicating a potential degree of cognitive flexibility in lexical processing (Johnston & Barry, 2006; Navarrete et al., 2015). The connection between acquiring a substantial vocabulary early on and improved retrieval efficiency further emphasizes the importance of developing a robust lexicon at a young age, which may have implications for enhancing cognitive resilience as individuals age (Catling & Johnston, 2009; Johnston & Barry, 2006; Navarrete et al., 2015).

Fluency appears to play an important role in the relationship between AoA and word retrieval performance. Enhanced fluency is often linked to improved lexical retrieval capabilities, suggesting that individuals with higher verbal fluency may handle TOT episodes more effectively than those with lower fluency levels (Cleary et al., 2021; Huebert et al., 2023). This potential connection suggests that, together with vocabulary size and AoA, fluency is a significant factor in alleviating the cognitive difficulties that often arise during word retrieval tasks (Frankenberg et al., 2021). Furthermore, a well-developed vocabulary may positively impact fluency, potentially providing a protective mechanism against age-related cognitive decline (Francis et al., 2021).

The interplay between fluid intelligence and verbal fluency might be crucial for understanding language retrieval (Frankenberg et al., 2021), especially in relation to the occurrence of TOT experiences. Fluid intelligence, which refers to the cognitive ability to solve novel problems and adapt to new situations, is recognized as important for various cognitive tasks, including language processing (Engle & Kane, 2004; Mitchell et al., 2023). Verbal fluency relates to an individual’s capacity to quickly generate words based on specific criteria, influenced not only by vocabulary availability but also by cognitive processes such as working memory and executive functioning (Roca et al., 2014). Research has suggested that those with higher fluid intelligence may exhibit better verbal fluency, enabling them to manage TOT states more efficiently than those with lower fluid intelligence (Mitchell et al., 2023; Salas et al., 2021).

This particular relationship is particularly relevant when considering the cognitive demands experienced during TOT moments. Individuals in a TOT state might find themselves struggling to retrieve a specific word, despite being confident they know it, indicating a disruption in retrieval mechanisms that may require the mobilization of cognitive resources to resolve the impasse (Frankenberg et al., 2021; Engle & Kane, 2004; Mitchell et al., 2023). Those with higher fluid intelligence are likely to utilize adaptive strategies more effectively (Martin et al., 2021; Tucker-Drob et al., 2022), possibly increasing their success in retrieving elusive words and alleviating frustration associated with TOT episodes. Additionally, research has indicated that fluid intelligence plays a vital role in allowing efficient transitions between cognitive tasks, which is essential during word retrieval as it aids in strengthening connections between phonological and semantic networks, thus enhancing overall retrieval performance. However, existing studies also confirm that as cognitive capabilities decline with age, individuals often experience reductions in both fluid intelligence and verbal fluency, which may increase the frequency of TOT experiences (Mitchell et al., 2023; Salas et al., 2021). The interaction between fluid intelligence and verbal fluency highlights the importance of understanding how fluid intelligence contributes to successful language recovery, particularly regarding the influence of verbal fluency on TOTs in older adults.

Beyond this relationship, the idea of crystallized intelligence adds another layer of understanding by connecting accumulated knowledge and vocabulary to improved outcomes in verbal retrieval (Hills, 2025). Crystallized intelligence, which tends to stabilize or increase with age, supports verbal fluency and seems to correlate with better performance on language production tasks. Individuals with higher levels of Gc are typically associated with a broader vocabulary and exhibit greater fluency in their expressions, which serve as a cognitive buffer during instances of TOT (Zaval et al., 2015). Consequently, those with elevated levels of Gc might experience fewer TOT episodes, reflecting the interactions between lexical knowledge, cognitive processing, and the complexities of retrieval tasks.

The evidence presented by Rojas et al. (2023a, 2023b) could support the notion that vocabulary (or crystallized intelligence) serves as a protective factor for cognitive health in older adults. Their findings suggest that lexical knowledge might remain relatively stable among the very old (80 years and older), potentially compensating for other aspects of cognitive aging (Riffo et al., 2020). Furthermore, an early acquisition of vocabulary seems to correlate with greater fluency, which might improve memory retrieval mechanisms, suggesting that it could have a modulatory effect on the frequency of TOT and FOK experiences.

### 1.3. The Present Study

In summary, the intricate connections between age of acquisition (AoA), verbal fluency and vocabulary size offer valuable insights into the cognitive mechanisms underlying word retrieval. However, a key research question remains: how do the interactions between AoA, verbal fluency and vocabulary affect the frequency of TOT and FOK episodes in very old age? This is important because previous studies have suggested that having a large vocabulary acquired early in life may improve cognitive performance in old age (Stern, 2009). In this sense, this research is justified by several key points. Firstly, older adults often report more frequent TOT experiences, which are commonly attributed to age-related decline in effective word retrieval (Brown, 2012). Therefore, it is essential to examine the role of AoA and vocabulary in these cognitive processes. Furthermore, this study aims to elucidate how improving language skills through increased vocabulary and fluency could positively impact the cognitive abilities of older populations. To explore these interactions, the study will conduct a TOT and FOK experiment incorporating methodological elements previously proposed by Gollan and Brown (2006). This experiment will control for the AoA of the stimuli while assessing participants’ vocabulary and verbal fluency.

It is hypothesized that older adults with an earlier AoA will experience fewer TOT and FOK episodes than those with a later AoA. Furthermore, individuals with a larger vocabulary and greater verbal fluency are anticipated to demonstrate a notable reduction in retrieval failures, emphasizing the significance of early vocabulary development in fostering optimal cognitive performance during ageing.

## 2. Materials and Methods

### 2.1. Participants

A total of 60 monolingual older adults willingly took part in this study. Recruiting participants who are 60 years old and above, especially those over 80, poses substantial difficulties owing to limitations related to health, mobility, and overall availability. Nevertheless, we managed to enroll 60 participants who satisfied rigorous inclusion and exclusion criteria. Although larger sample sizes are typically advantageous for boosting statistical power, research targeting these older age groups frequently relies on smaller samples, largely attributed to similar recruitment challenges (e.g., Artuso & Belacchi, 2021; Martín-Aragoneses et al., 2023; Rojas et al., 2023a). All older adults were recruited through the “Más Adultos Mayores Autovalentes (More Self-Sufficient Seniors)” program, which is sponsored by the Chilean government. The inclusion criteria for participation were as follows: participants had to be at least 60 years old, possess a minimum of six years of formal education, exhibit normal (or corrected) hearing and vision, reside in urban areas, and complete the reading comprehension task (subitem of the Abbreviated Boston Test) within a maximum of two weeks. Descriptive demographic statistics of our sample are detailed in Table 1.

Additionally, it was necessary to maintain current medical records for participants to confirm that they were in good health. We established specific exclusion criteria, which included: a history of cerebrovascular disease, a diagnosis of neurodegenerative disorders, the presence of depression or other psychiatric conditions, and a risk score on any of the following psychometric assessments: Mini-Mental State Examination (MMSE score < 23 points; Folstein et al., 1975) or Geriatric Depression Scale-15 (score > 11 points; Martínez de la Iglesia et al., 2002). Approximately 100 older adults were invited to participate in the study. We determined a sample size capable of yielding at least 1500 data points. Among those expressing interest in participation, older adults who did not meet the set inclusion and/or exclusion criteria were subsequently excluded from the study. To take part in this study, all older participants were required to read and sign an informed consent form that had been approved by the Ethics Committee of the sponsoring university (cod. 11230984). The study’s objectives and details were communicated to the authorities of each associated university senior club. Subsequently, older adults expressing interest in participation underwent assessments evaluating their cognitive performance using the Mini-Mental State Examination (MMSE), as well as their emotional well-being via the Geriatric Depression Scale-15. Ultimately, the selected participants were invited to the university’s Language and Cognition Laboratory to take part in the experiment and complete the vocabulary and verbal fluency tests.

### 2.2. Materials and Design

The TOT/FOK experiment is grounded in the two-step approach proposed by Gollan and Brown (2006), which posits that spoken word production involves two essential stages of retrieval: the first stage pertains to meaning-based lexical selection, while the second stage involves form-based word-form encoding (Levelt et al., 1999). Gollan and Brown (2006) developed a framework to estimate the probabilities of semantic and phonological retrieval failures during instances of the TOT phenomenon. According to their methodology, according to their methodology, responses are categorized into five distinct types that indicate varying degrees of success or failure across the two retrieval stages. A “Got the words” (GOT) response signifies successful retrieval in both steps, whereas a positive TOT indicates successful semantic retrieval but failure in phonological retrieval. Conversely, a negative TOT reflects failure in both semantic and phonological retrieval processes. The participants may also offer a “not GOT” response, where they can recognize the correct target word yet are unable to retrieve it upon initial attempt. Additional responses include “don’t know” (DK) or “post-don’t know” (post-DK) regarding their recognition capabilities. By employing this two-step approach, Gollan and Brown (2006) quantitatively analyzed the relationship between aging and its detrimental effects on phonological retrieval relative to semantic retrieval, specifically for target words deemed difficult. This method has been tested in various contexts, revealing the influence of different lexical variables and the cognitive conditions of participants on word retrieval. The effectiveness of this approach demonstrates its sensitivity to the complexities of spoken word production, providing a more detailed insight into the challenges people face with lexical access as they age (Bannon et al., 2024; Medeiros, 2024).

In this experiment, images depicting both early-acquired and late-acquired words were utilized. This methodological choice distinguishes our study from other research that frequently employs faces or proper names. By focusing on real and inanimate objects (and the vocabulary they elicit), as opposed to faces associated with proper names, it is possible to control the variable of age of acquisition more precisely. This differentiation is crucial, as it allows us to investigate how the variable of AoA influences word retrieval processes and contributes to the occurrence of TOT and FOK states. Previous studies have shown that early-acquired words tend to yield faster and more reliable retrieval responses compared to late-acquired words, further emphasizing the relevance of AoA in language production (Cleary & Claxton, 2015). By using object images, our experiment capitalizes on the advantages of clear and concrete stimuli, which enhance the detectability of retrieval difficulties and the resulting TOT or FOK experiences, thereby elucidating the intricate relationship between the timing of word acquisition and the cognitive processes involved in spoken word retrieval.

To ensure the appropriate selection of images for the experiment, a normative study was conducted. In this study, a total of 200 images were chosen, consisting of 100 representing early-acquired words and 100 corresponding to late-acquired words, based on a consensus established among ten professional speech-language pathologists. Subsequently, these 200 images were evaluated by a normative group comprising ten older adults. Additionally, it is important to note that the Likert scale used in the normative study ranged from 1 to 10. A score of 1 indicated a word acquired in the early stages of life or early childhood (before the age of three). A score of 5 was equivalent to a word acquired during middle childhood (ages 4–5). A score of 10 was for more complex words acquired during the school years (aged 6 and over). Based on this criterion, all words with a score below 4 were classified as having been acquired early. Words with a score above 5 were classified as late acquisition. This rigorous selection process resulted in an experimental set comprising 100 words, evenly divided into 50 early-acquired and 50 late-acquired items, along with an additional 30 neutral filler objects representing medium-age acquisitions (obtained from the initial normative study). This careful methodology enhances the reliability of the experimental outcomes by ensuring that the stimuli are appropriately aligned with the research objectives regarding the influence of age of acquisition on word retrieval and associated TOT and FOK states.

Regarding the tests administered, first, the Boston Aphasia Diagnostic Test (BADT), developed by Goodglass and Kaplan (1983), is a recognized tool for assessing language function in individuals suspected of having aphasia. The test comprises several components that focus on various verbal abilities, including vocabulary. The vocabulary subtest evaluates an individual’s ability to retrieve and produce words, measuring aspects such as naming capacity and verbal comprehension. Within the BADT vocabulary test, clinicians utilize various methods to assess word retrieval skills. One such method is a confrontation naming task, in which participants are shown pictures and asked to name the depicted objects. The test also evaluates verbal tasks related to word production under phonemic and semantic constraints, reflecting the cognitive processes underlying language production (Martielli & Blackburn, 2016). Paying close attention to naming accuracy and response time is essential for diagnosing specific language impairments and for developing personalized therapeutic strategies.

Another essential component of the assessment was the verbal fluency test (Lezak, 2004), consisting of two main types of tasks: phonemic and semantic fluency. In the phonemic fluency test, individuals generate as many words as possible starting with a specific letter (i.e., ‘F’, ‘A’ or ‘S’) within one minute. Performance on these tasks indicates executive function, lexical access and retrieval speed, all of which can be impaired in various types of diagnostics. The semantic fluency test requires participants to list words belonging to a particular category, such as animals or fruits, within one minute. This task assesses the ability to retrieve semantically related concepts.

### 2.3. Apparatus

The TOTs-FOKs experiment was conducted utilizing E-Prime Professional Software 3.0. Images were displayed on a high-resolution monitor with a resolution of 1024 × 768 pixels. Participants’ responses were captured using a microphone connected to an Asus-compatible PC via a PST Serial Response Box (Chronos voice key).

### 2.4. Procedure

The experiment was conducted in a private room that was well-lit and acoustically treated to minimize external noise. Visual stimuli were presented prominently in the center of a 15.6-inch computer screen. Participants read the on-screen instructions, with sufficient opportunity provided for clarification and questions. The instructions outlined the following scenarios: (1) “At times, you will clearly and effortlessly know the name of the image”; (2) “On other occasions, you may have a strong feeling of knowing the name, as if it is on the tip of your tongue”; (3) “In some instances, you might feel a familiarity with the image but be unable to remember the name at all, indicating that it is not on the tip of your tongue”; (4) “Finally, it is possible that you simply do not recognize the presented image at all.”

In the subsequent display, participants were informed: “In this task, you will make your decision by pressing a button.” The instructions emphasized, “There are no right or wrong answers.” The specific response options included: (1) If you know the image and can effortlessly state the word, press the green button (I know); (2) If you have the sensation of it being on the tip of your tongue but require effort to recall, press the yellow button (TOT); (3) If you feel familiar with the image, believe you know it, but cannot remember the name, press the blue button (FOK); (4) If you do not know or recognize the image, press the red button (I do not know).

**Figure 1:**
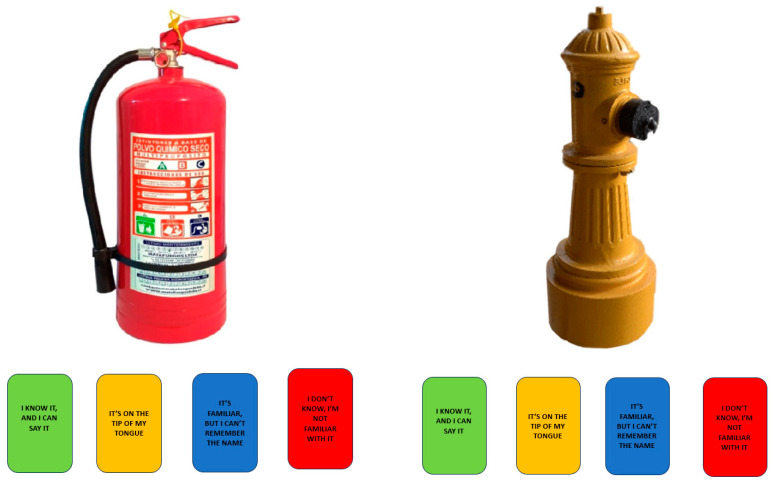
Examples of experimental trials with options of response (colored buttons).

Once participants comprehended the task requirements, they proceeded to the training stimuli. The main task commenced with a fixation point displayed for 500 milliseconds, accompanied by a click sound. Following this, the image appeared at the top of the screen, while at the bottom, the four response buttons were displayed alongside their corresponding instructions (see Figure 1). Participants were allocated 5000 milliseconds to respond; if they did not provide a response within this timeframe, the experiment progressed to the subsequent trial. If the participant pressed the green button (indicating “I know”), a new display prompted, “Now say the word.” At this point, the Chronos voice key was activated to record the response, allowing for the measurement of reaction times.

**Figure 2:**
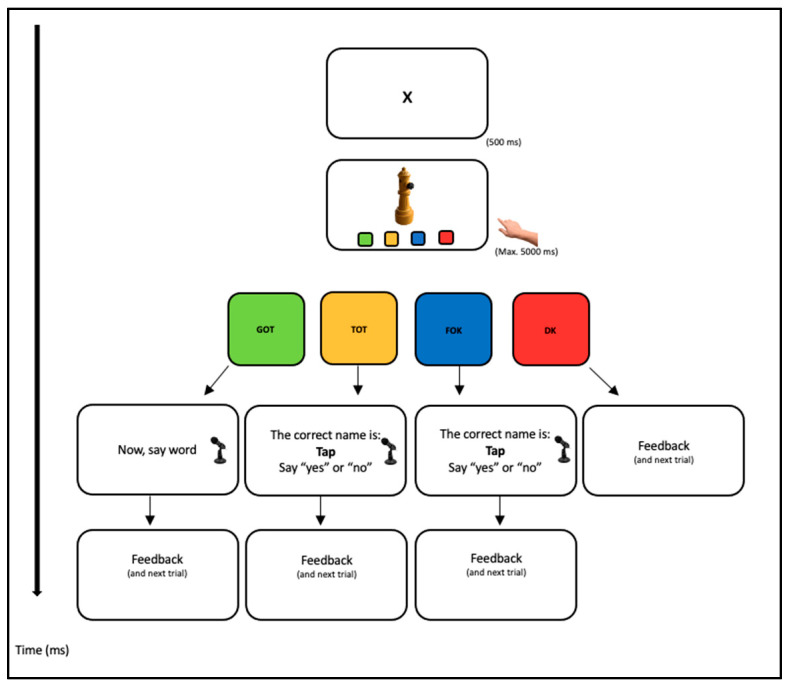
Illustrate the details of the procedure and the configuration of each experimental test.

Should the yellow button (indicating TOT) be pressed, the next display would show the message, “The correct name is xxx; yes or no?” In this experiment, only accurate answers were provided. The voice key would then record the participant’s response. The procedure for pressing the blue button (FOK) mirrored that of the yellow button for TOT responses. Lastly, if the red button was pressed, the following slide displayed the message, “No problem, let’s move on to the next one.” If participants did not respond within 5 s, the experimenter would initiate the next trial (5 s corresponds to the time during which the figure is displayed and the participant’s response is awaited). The Chronos voice key enabled the E-Prime software to accurately monitor the time elapsed from stimulus presentation to the participant’s oral response, facilitating the calculation of reaction times for each trial. The experiment was organized into two blocks, separated by a brief intermission, with the entire session lasting approximately 25 min (the experiment will be available upon request to the lead author by email). For more details on the procedure, see Figure 2.

### 2.5. Data Analysis

The statistical analysis was conducted using regression models that incorporated cross-mixed effects. This analysis used the lme4 (Bates et al., 2015) and lmerTest (Kuznetsova et al., 2017) packages in the R statistical software project. These packages allow for the inclusion of intrinsic variability at both the participant and item levels within a single regression framework, thus eliminating the need for additional data aggregation. After computing descriptive statistics (mean and standard deviation) and ensuring appropriate distribution adjustments, generalized linear models were employed to analyze the probabilities of episodes encompassing both TOT and FOK phenomenon. Data preparation entailed that each TOT or FOK episode was coded as 1, while trials with no episodes were coded as 0, resulting in a binomial distribution. Initially, we aimed to analyze TOT and FOK episodes separately. However, given the small percentage of episodes (7.8% and 1.1%, respectively), we decided to analyze them together (I know or I don’t know responses were excluded from this analysis). The response time for TOT + FOK was analyzed using linear models, given their continuous and log-normal distribution nature. The fixed effect structure of the models included participants’ sex as a discrete predictor, as well as their age, vocabulary and verbal fluency as continuous predictors. Finally, both models also incorporated AoA for each item, as well as two-way interaction terms between sex and AoA and vocabulary and verbal fluency variables. Both discrete predictors were sum-contrast coded (e.g., −1,1) for each level. All continuous predictors were scaled and centered using the scale () function in R. The random structure of the models included random intercepts for both participants and items, as well as random slopes for AoA for participants, and sex, age, vocabulary and verbal fluency as random slopes for items. Data and script files can be found in our OSF repository https://osf.io/tk7sz/overview?view_only=9633445598fb46cfb5aadee96cc5c6c2 (accessed on 10 October 2025). Finally, to access the list of words used, the normative study scores, and the scores from the verbal fluency and vocabulary tests administered, see Supplementary Material.

## 3. Results

Table 2 illustrates the results from a logistic regression analysis investigating the probability of experiencing episodes of TOT and the FOK phenomenon. Using the *vif()* function from the *car* R package (version 4.4.1) we determined that the model presented no collinearity, as all vif values were below 5 (see Hair et al., 2010). The model’s intercept is estimated at a probability of approx. 1.7% (logarithmic probability ratio = −4.06) and significantly different from 0. This intercept reflects the probability of experiencing TOT or FOK episodes when all predictors are set to zero (or to the mean in the case of centered continuous predictors), indicating a quantifiable probability of these occurrences within the studied population.

Among the fixed effects examined, age of acquisition (AoA) emerges as a significant predictor. This finding emphasizes the critical role of AoA in cognitive performance, particularly in relation to language recovery. It suggests that words learned later in life lead to more TOTs and FOKs in this context, reflecting the cognitive advantages associated with early language learning.

Conversely, the standardized age variable demonstrates no statistical significance, indicating that variations in age do not independently predict the probability of experiencing TOT or FOK within this sample. Similarly, the sex variable, from an exploratory perspective, failed to reach significance, suggesting that gender does not impact the likelihood of these cognitive phenomena in this context.

**Figure 3:**
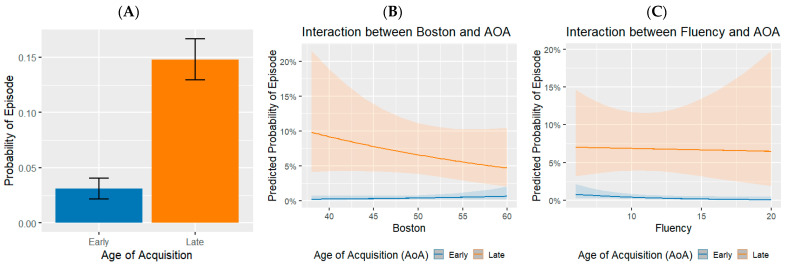
Probability of reporting an episode across predictors. Panel (**A**) shows the mean probability as a function of AoA. Panels (**B**,**C**) display the estimated effects of vocabulary and verbal fluency, respectively, on that probability. The ribbon area around the lines represents CI95% confidence intervals.

The analysis also reveals significant interaction effects among predictors. Notably, the interaction between AoA and vocabulary size indicates that the relationship between AoA and the probability of TOT or FOK episodes diminishes as vocabulary strength increases (see Figure 3). This finding implies that individuals with a more extensive vocabulary may rely less on AoA during word retrieval, supporting the hypothesis that richer lexical knowledge facilitates cognitive retrieval.

Additionally, the interaction between AoA and verbal fluency is significant, demonstrating that those with higher fluency experience less impact from the age of word acquisition on retrieval likelihood (see Figure 3). This highlights fluency as a protective factor enhancing cognitive resilience, as individuals who demonstrate greater fluency can navigate TOT episodes more effectively.

Table 3 presents the results of a fixed effects analysis examining how predictors influence RT during lexical retrieval. Among the predictors analyzed, AoA stands out, yielding a significant effect. This result affirms the substantial impact of AoA, suggesting that earlier vocabulary acquisition is associated with quicker RT—demonstrating the cognitive advantages of early learning. Again, for this model, we observed no collinearity.

**Figure 4:**
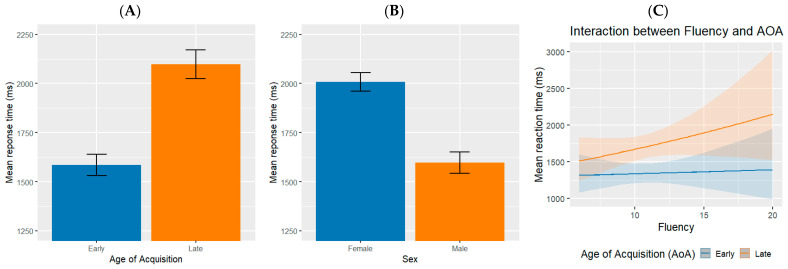
Panel (**A**) shows the mean reaction time as a function of age of acquisition, Panel (**B**) shows the mean reaction time as a function of participants’ sex, and Panel (**C**) shows the mean reaction time as a function of age of acquisition and verbal fluency. The ribbon area around the lines in Panel (**C**) represents CI95% confidence intervals.

The analysis of age reveals no significant impact on RT. Additionally, verbal fluency and vocabulary size did not appear as significant on response latencies, indicating that these factors alone may not be critical determinants in this context. Conversely, at an exploratory level, the sex variable shows a significant effect on RT. This implies that females tend to have longer RT relative to males. Finally, the notable interaction between AoA and fluency indicates that individuals who show greater fluency benefit more from earlier-acquired vocabulary, leading to faster retrieval times. This result suggests that fluency may enhance the retention of cognitive retrieval pathways developed during early learning (see Figure 4).

## 4. Discussion

The research aimed to investigate the TOT and FOK phenomena in older adults, which are characterized by difficulties in retrieving words that one believes they know, often accompanied by frustration (Barry et al., 2025; Kim & Choi, 2021). The findings reveal significant insights into the interconnections between age of acquisition (AoA), vocabulary size, and verbal fluency with regard to TOT and FOK experiences. Older adults who acquired vocabulary earlier experienced fewer instances of TOT and FOK, suggesting that early language exposure promotes better lexical access in later life (Brysbaert & Ghyselinck, 2006; Elsherif et al., 2023; Johnston & Barry, 2006). The analysis corroborates the idea that vocabulary size and verbal fluency play critical mediating roles in the decline of cognitive processing with age: those with broader vocabularies and higher fluency demonstrated improved efficiency in navigating lexical retrieval tasks (Catling & Johnston, 2009; Johnston & Barry, 2006; Navarrete et al., 2015). Furthermore, statistical analyses revealed differences in RT between male and female participants, though the implications of these discrepancies require further investigation in light of the conflicting literature (Goral et al., 2007). Phonological factors also emerged as important, with older individuals experiencing an increased number of TOT occurrences due to reduced phonological accessibility. This suggests that retrieval failures may reflect weakened associations between word meanings and sound structures developed earlier in life (Navarrete et al., 2015; Salthouse & Mandell, 2013).

A significant body of the literature has explored the construct of AoA in relation to TOT and FOK experiences. Earlier vocabulary acquisition correlates positively with retrieval efficiency, implying that individuals who learn words earlier tend to have better access to these words later in life (Rojas et al., 2023a). This relationship underscores the protective effects of early language exposure against cognitive decline associated with aging. Research indicates that AoA significantly enhances lexical retrieval and reduces the likelihood of experiencing TOT and FOK phenomena, reinforcing the idea that a rich early vocabulary lays a foundation for better cognitive outcomes during advanced aging (Mitchell et al., 2023; Salas et al., 2021).

Moreover, vocabulary size and verbal fluency play essential roles in the dynamics of TOT and FOK experiences. Studies reveal that individuals with extensive vocabularies and high verbal fluency skills exhibit greater resilience against age-related declines in cognitive processing, positively influencing their retrieval abilities (Frankenberg et al., 2021; Engle & Kane, 2004; Mitchell et al., 2023). Specifically, older adults with strong vocabulary resources navigate through TOT states more efficiently, with a smaller effect of AoA as a determinant of retrieval success, suggesting a nuanced interplay between these variables.

In this sense, fluid intelligence introduces an additional layer of complexity, as it influences cognitive performance across various tasks, including language processing and verbal fluency. Fluid intelligence plays a key role in determining how individuals access their lexical resources, especially as they age (Mitchell et al., 2023). In contrast, crystallized intelligence, which encompasses accumulated knowledge and skills, including vocabulary, plays a pivotal role in maintaining verbal fluency in older adults. While fluid intelligence typically declines with age, crystallized intelligence remains relatively stable, enabling older adults to utilize their extensive vocabulary effectively during verbal tasks (Rojas et al., 2023a; Mitchell et al., 2023). This stability is particularly relevant as it can mitigate the decline in access to lexical items, as evidenced by the increased occurrence of TOTs in ageing populations (Gertel et al., 2020; Gollan & Brown, 2006). Understanding how these two forms of intelligence interact provides insight into the modulation of lexical access, where both fluid/crystalized intelligence contribute to language processing efficiency. In older adults, robust crystallized intelligence supports retrieval during TOT states, thereby enhancing vocabulary usage and overall communication efficacy (Mitchell et al., 2023). Consequently, the interplay of fluid and crystallized intelligence is essential for modulating how older adults access their lexicon, influencing the frequency and severity of TOTs and FOKs experienced.

In examining the nuances of RT across genders, the analysis reveals significant distinctions between males and females in the context of the TOT and FOK phenomena. The results indicate that, under certain conditions, males may demonstrate faster reaction times than their female counterparts, particularly in the realm of lexical processing. The statistically significant difference reported implies that males may process and retrieve lexical items more efficiently than females. Prior research has chiefly illustrated a tendency for males to maintain quicker reaction times in diverse experimental settings (Lindenberger & Reischies, 1999). However, it is essential to examine the subtleties associated with specific cognitive tasks, as some studies present evidence that females may excel in certain retrieval tasks, potentially due to their cognitive processing strengths (Goral et al., 2007).

It is essential to consider phonological factors in the manifestation of TOT states. Research indicates that the phonological accessibility of words deteriorates with age, leading to a greater incidence of TOT experiences in older adults (Abrams & Davis, 2016; Brown, 2012; Ouyang et al., 2020). This phonological decline highlights the intricate connection between word meanings and their sound structures, suggesting that retrieval failures may arise due to weakened associations between these components, challenging cognitive pathways developed earlier (James & Burke, 2000). Evidence further supports that phonological characteristics significantly contribute to the likelihood of experiencing TOT during retrieval attempts, particularly when individuals have formed robust phonological representations in their early language development (Gollan et al., 2011).

The interplay of vocabulary size and fluency with AoA emerged as critical themes in the results. Individuals demonstrating higher vocabulary levels and fluency experienced fewer TOT episodes, underscoring a compensatory mechanism that arises from robust lexical knowledge. The observed interactions suggest that the contribution of AoA diminishes as vocabulary strength increases, reiterating the importance of vocabulary development across the lifespan for positively impacting cognitive retrieval processes.

By focusing on the cognitive dynamics at the intersection of AoA, vocabulary proficiency, and age-related cognitive changes, researchers gain significant insights into language processing. The findings suggest that interventions aimed at preserving and enhancing vocabulary size and verbal fluency reduce retrieval failures in older adults and improve their overall quality of life (Kavé & Sapir-Yogev, 2020). These insights have implications for healthcare strategies and educational programs that support early language acquisition, consistent with cognitive development and emotional well-being as individuals confront aging challenges.

As we look ahead, the implications of this study extend to both cognitive science and practical applications in gerontology. Understanding cognitive mechanisms underlying language retrieval in older adults is critical for developing interventions that promote effective communication and cognitive health (Abrams & Davis, 2016; Schwartz, 2010; Schwartz & Cleary, 2016). Therefore, fostering rich linguistic environments during early childhood is crucial for enhancing lifelong cognitive resilience. Additionally, the interactions between vocabulary fluency, AoA, and TOTs experiences indicate that cognitive decline may not uniformly affect all individuals. Those with robust vocabulary and higher fluency may experience these phenomena less frequently, suggesting a framework for targeted cognitive interventions that leverage these protective factors.

In evaluating the current research within the broader context of TOT and related cognitive phenomena, the findings pave the way for exploring how various lexical and cognitive attributes interact in older adults facing increased retrieval difficulties. Evidence from prior studies supports the need for further understanding of the cognitive processes underpinning TOT experiences, linking them to neuropsychological frameworks and practical applications within gerontology. Continued research in this area may yield significant insights into strategies for mitigating cognitive decline, fostering resilience, and ultimately enhancing the well-being of older adults as they navigate language retrieval challenges.

### Limitations and Future Perspectives

Despite its strengths, the study contains limitations, notably the small sample size, which may limit findings’ generalizability and robustness. The lack of assessments for other cognitive variables, such as processing speed and working memory, may also limit the understanding of the dynamics between TOT occurrences and cognitive decline. Incorporating larger and more diverse samples, as well as additional cognitive evaluations, could enhance future research outcomes.

First, understandinging cognitive phenomena such as TOT and FOK requires consideration of the overlap between lexical frequency and age of acquisition (AoA). Words learned earlier in life are more likely to be used frequently in everyday conversation and have been engaged with for a longer period in a speaker’s memory (Brysbaert & Ghyselinck, 2006). Consequently, this prolonged exposure enhances the accessibility of such words, making them more likely to be identified as ‘known’ during retrieval tasks. While our findings suggest that an earlier AoA is linked to more successful lexical retrieval, it is important to distinguish these effects from those of lexical frequency (Catling & Johnston, 2009; Elsherif et al., 2023). Future research should aim to control for word frequency to ascertain whether words acquired at similar frequency levels still yield different retrieval outcomes based on AoA. This would provide a clearer understanding of the cognitive processes underlying TOT and FOK experiences, and further elucidate the relationship between early vocabulary exposure and cognitive processes in ageing populations.

Furthermore, we need to report on the potential ceiling and floor effects related to verbal fluency and vocabulary size in our study. In this regard, it is important to recognize that words acquired at an early stage tend to be used more frequently in everyday conversation, which can result in upper limits of performance in TOT and FOK responses. This can obscure the nuanced effects of vocabulary size and fluency on retrieval performance. Conversely, words acquired later in life tend to demonstrate a wider range of performance levels. This enables individuals with a more extensive vocabulary and greater verbal fluency to demonstrate their abilities more effectively when retrieving these less familiar words. This distinction highlights the importance of carefully considering both AoA and lexical frequency when analyzing cognitive retrieval processes. A greater understanding of how these elements interact will facilitate more comprehensive findings on their respective impacts on TOT and FOK experiences.

The methodology used to select words acquired early versus late raises important considerations about the reliability of categorization processes. Although the study used a normative sample of ten normal older adults, the limitations of such a small sample size deserve attention. Relying solely on subjective assessments, rather than conducting reliability tests, may have introduced biases in classifying words according to their age of acquisition. Furthermore, it is important to recognize that individuals acquire words at different stages of language development, adding complexity to the interpretation of the results. Future research could benefit from using a larger normative sample or objective measures alongside subjective assessments to ground word classifications more robustly and account for variability in individual acquisition experiences.

Another limitation is the observed age difference between male and female participants in this study. This difference is mainly due to the recruitment method, which included the Chilean government’s Más Adultos Mayores Autovalentes (More Self-Sufficient Older Adults) programme. This programme predominantly attracts younger older women, aged 60–70, while older men and women are underrepresented. Consequently, it was difficult to match participants of both sexes by age due to this demographic bias. To mitigate the potential effects of this age disparity on study outcomes relating to the TOT and FOK phenomena, robust statistical controls were employed. Specifically, our regression models incorporated participant age as a continuous variable and gender as a discrete variable, enabling a nuanced examination of their individual and interactive effects on cognitive outcomes. This rigorous analytical approach ensures the reliability of our results despite the variability introduced by the age differences.

Future research iterations have opportunities for enhancement by increasing sample sizes to improve data robustness and generalizability. A longitudinal design could provide insights into the evolution of TOT and FOK experiences over time. Expanding vocabulary evaluations to include subjective word frequency and familiarity measures would yield a better understanding of how individual differences affect retrieval challenges. Additionally, assessing participants’ confidence regarding their retrieval capabilities could capture metacognitive aspects of TOT experiences, enriching perspectives on cognitive states during language processing.

The findings carry significant implications for cognitive science and gerontology, especially as the population ages. Understanding the cognitive mechanisms of language retrieval in older adults is vital for developing effective interventions. Results indicate that early vocabulary acquisition serves as a protective factor against aging-related cognitive challenges, highlighting the importance of fostering rich linguistic environments in childhood to enhance lifelong cognitive resilience. The study underscores that cognitive decline does not uniformly affect individuals; those with robust vocabularies and higher fluency tend to experience fewer TOT occurrences. Thus, targeted cognitive interventions focusing on vocabulary enrichment and verbal fluency training in later life may significantly mitigate age-related cognitive decline, thereby improving the quality of life for older adults facing communication challenges. Overall, the study emphasizes the crucial interplay of AoA, vocabulary size, and verbal fluency in cognitive processes associated with TOT phenomena among older adults, offering insights essential for fostering cognitive health within aging populations.

## 5. Conclusions

The present study highlights the significant interplay between AoA, vocabulary size, and verbal fluency in influencing the occurrences of the TOT and FOK phenomena among older adults. The results demonstrate that earlier vocabulary acquisition is a strong predictor of enhanced lexical retrieval efficiency, suggesting a protective factor against cognitive decline associated with aging. Specifically, individuals with larger vocabularies and greater verbal fluency exhibit fewer TOT episodes, emphasizing the importance of robust lexical knowledge in mitigating retrieval challenges.

Moreover, the findings underscore that while cognitive aging impacts language processing, factors such as AoA and vocabulary proficiency can significantly buffer these effects. The study advocates for educational and clinical interventions focused on enhancing vocabulary and fluency from an early age and throughout life. Such initiatives hold promise for improving the quality of life for older adults, facilitating effective communication and cognitive health as they navigate the challenges associated with aging. Our research contributes to a deeper understanding of the cognitive mechanics underlying language retrieval and establishes a foundation for future studies aimed at developing targeted strategies to support linguistic competency in older populations.

## Figures and Tables

**Table 1:** Demographic statistics of study group.

Sex	N	Age		Schooling		MMSE		GDS-15	
		Mean/Sd	Min/Max	Mean/Sd	Min/Max	Mean/Sd	Min/Max	Mean/Sd	Min/Max
Female	35	71.05/7.75	60/87	9.85/0.42	6/12	26.90/1.37	24/30	14.23/0.87	12/15
Male	25	81.17/6.49	63/88	10.27/0.93	6/12	27.20/0.97	24/30	13.59/1.21	12/15
Total	60	75.66/8.79	60/88	10.06/0.59	6/12	27.05/1.17	24/30	13.91/1.04	12/15

**Table 2:** Logistic regression analysis for the variables of age, sex, AoA, vocabulary and verbal fluency on probability of episode (TOT + FOK).

Variable	Estimate	Std. Error	z Value	Pr(>z)
(Intercept)	−4.059234	0.293551	−13.828	<0.001 *
Age	−0.121617	0.263623	−0.461	0.645
Sex	0.263027	0.210190	1.251	0.211
AoA	1.491063	0.211811	7.040	<0.001 *
Vocabulary	−0.002958	0.174873	−0.017	0.987
V. Fluency	−0.361272	0.260287	−1.388	0.165
Sex: Vocabulary	−0.218986	0.178029	−1.230	0.219
AoA: Vocabulary	−0.211448	0.093648	−2.258	0.024 *
Sex: V. Fluency	−0.218715	0.175873	−1.244	0.214
AoA:V. Fluency	0.301563	0.125148	2.410	0.016 *

* = *p* < 0.05.

**Table 3:** Mixed linear regression (log) for the variables of age, sex, AoA, vocabulary and verbal fluency on RT (TOT + FOK episodes).

Variable	Estimate	Std. Error	t Value	Pr(>t)
(Intercept)	7.294517	0.045250	161.206	<0.001 *
Age	0.041222	0.076048	0.542	0.589
Sex	−0.167433	0.056582	−2.959	0.004 *
AoA	0.120437	0.017078	7.052	<0.001 *
Vocabulary	0.004687	0.047737	0.098	0.922
V. Fluency	0.056234	0.069709	0.807	0.423
AoA: Vocabulary	−0.011894	0.009551	−1.245	0.218
AoA: V. Fluency	0.040841	0.010066	4.057	<0.001 *

* = *p* < 0.05.

## Data Availability

Data available upon request. Please email the lead author.

## Supplementary Materials

The following supporting information can be downloaded at: https://www.mdpi.com/article/10.3390/bs15121686/s1, (1) Verbal fluency and vocabulary tests administered; (2) Vocabulary results - Verbal fleuncy (N = 60); (3) Trial list (Scores Normative AoA).
