# Supplementary material for: Exploring the “Tip of the Tongue” and “Feeling of Knowing” Phenomena During Advanced Aging: The Interplay of Age of Acquisition, Vocabulary and Verbal Fluency
Source: Behav Sci (Basel). 2025 Dec 5;15(12):1686. doi: 10.3390/bs15121686 (PMC12729277; doi:10.3390/bs15121686)
Supplement: Supplementary file 1 [file behavsci-15-01686-s001.zip › behavsci-3991465-supplementary.pdf]

Cuadernillo de Respuesta
Fluidez Verbal y Vocabulario

PROYECTO FONDECYT 11230984

Nombre: **Edad:**

I. Test de Fluidez verbal fonológica y semántica

Descripción: En esta tarea se solicita una búsqueda verbal de ítems de tres categorías fonológicas (F, A ,S) y tres categoría semánticas (animales, cosas y alimentos) durante un tiempo determinado.

Aspectos a tener en cuenta: - Cronometrar 60 segundos para cada parte.

- Se deben anotar en letra legible todas las palabras en el orden que lo dice el evaluado, incluyendo las perseveraciones e intrusiones. Puede usar una grabadora para apoyarse.

Aplicación Instrucción: "En esta tarea usted va a tener un minuto para decirme todas las palabras que se le ocurran que comiencen por una letra que más adelante le voy a decir. Puede decir todas las palabras que empiecen por esa letra excepto nombres propios (personas, lugares, marcas) o variaciones de la misma palabra. Por ejemplo, si fuera la letra D no me podría decir Dolex, Diego, Duarte, ni Dinamarca porque son nombres propios; ni tampoco dedo, dedito, dedote. ¿Es claro? Para la tarea de fluidez verbal semántica se da la siguiente instrucción: "Va a repetir la tarea, pero esta vez quiero que me diga todos los animales que recuerde, sin importar la letra por la que empiezan, a partir de ya"

	F	A	S	Animales	Cosas	Alimentos
1						
2						
3						
4						
5						
6						
7						
8						
9						
10						
11						
12						
13						
14						
15						
16						
17						
18						
19						
20						
	**Total:**	**Total:**	**Total:**	**Total:**	**Total:**	**Total:**

II. Test de Vocabulario de Boston

Descripción: Consta de 3 láminas, cada una de las cuales contiene 100 elementos.

El test de vocabulario del Boston consta de 60 figuras, ordenadas desde la más fácil a la más difícil. Las figuras se presentan en orden, permitiendo un tiempo de 20 segundos al sujeto para que responda.

Las puntuaciones que proporciona la prueba son:

- El número de respuestas correctas emitidas espontáneamente.
- El número de claves semánticas dadas por el examinador. Por ejemplo: la clave semántica de la figura "cama" es "un mueble".
- El número de respuestas correctas después de una clave semántica proporcionada por el examinador.
- El número de claves fonéticas dadas por el examinador (al paciente se le proporciona el sonido inicial de la palabra).
- El número de respuestas correctas después de una clave fonética proporcionada por el examinador.

Nº	Ítem	Respuesta	Correcta	Clave Semántica (si-no)	Correcta con CS	Clave Fonológica (si-no)	Correcta con CF
1	**Cama** (un mueble)						
2	**Árbol** (crece en el campo)						
3	**Lápiz** (sirve para escribir)						
4	**Casa** (un tipo de edifico)						
5	**Reloj** (sirve para ver la hora)						
6	**Tijeras** (sirve para cortar)						
7	**Peine**(arreglarse el pelo)						
8	**Flor** (crece en el jardín)						
9	**Hacha** (uso carpintero)						
10	**Sacapunta** (saca punta al lápiz)						
11	**Helicóptero** (viajar por el aire)						
12	**Escoba** (sirve para limpiar)						
13	**Pulpo** (un animal marino)						
14	**Zanahoria** (algo para comer)						
15	**Percha** (está en el armario)						
16	**Termómetro** (mide temperatura)						
17	**Camello** (un animal)						
18	**Máscara** (parte de un disfraz)						
19	**Queque** (algo para comer)						
20	**Banco** (sirve para sentarse)						
21	**Raqueta** (se utiliza en un deporte)						
22	**Caracol** (un animal)						
23	**Volcán** (un tipo de montaña)						
24	**Pez espada** (animal marino)						
25	**Dardo** (sirve para lanzar)						
26	**Canoa** (se usa en el agua)						
27	**Globo** (un tipo de mapa)						
28	**Corona** (la usan los reyes)						
29	**Castor** (un aminal)						
30	**Armónica** (instrumento musical)						
31	**Rinoceronte** (un animal)						
32	**Bellota** (proviene de un árbol)						
33	**Iglú** (un tipo de casa)						
34	**Zancos** (para caminar más alto)						
35	**Dominó** (un juego)						
36	**Cactus** (algo que crece)						

37	**Escalera mecánica** (para subir)						
38	**Arpa** (instrumento musical)						
39	**Hamaca** (sirve para descansar)						
40	**Cerradura** (para abrir la puerta)						
41	**Pelícano** (un ave)						
42	**Fonendoscopio**(uso de médicos)						
43	**Pirámide** (está en Egipto)						
44	**Bozal** (se utiliza para los perros)						
45	**Unicornio** (animal mítico)						
46	**Embudo** (para verter un líquido)						
47	**Acordeón** (instrumento musical)						
48	**Aguja** (se usa para coser)						
49	**Espárrago** (algo para comer)						
50	**Compás** (sirve para dibujar)						
51	**Chupete**(lo usan los bebés)						
52	**Trípode** (lo usan los fotógrafos)						
53	**Pergamino** (un documento)						
54	**Pinzas** (un utensilio)						
55	**Esfinge** (se encuentra en Egipto)						
56	**Yugo** (para animales de tiro)						
57	**Regadera** (se usa en el jardín)						
58	**Paleta** (la usan los artistas)						
59	**Transportador** (medir ángulos)						
60	**Ábaco** (sirve para contar)						
	**TOTALES**						

RESULTS VOCABULARY - VERBAL FLUENCY (N=60)

Participant	Sex	Age	Vocabulary	V. Fluency
1	Female	71	40	13,0
2	Female	62	43	13,4
3	Female	71	41	9,7
4	Female	65	46	17,0
5	Female	62	54	17,2
6	Female	75	46	14,7
7	Female	65	52	14,2
8	Female	76	44	10,7
9	Female	67	50	12,4
10	Female	70	48	15,9
11	Female	74	48	13,3
12	Female	61	42	15,0
13	Female	69	44	11,7
14	Female	60	48	14,0
15	Female	61	41	14,4
16	Female	68	58	11,7
17	Female	67	44	18,2
18	Female	76	43	11,8
19	Female	68	46	10,7
20	Female	68	39	9,3
21	Female	68	48	20,0
22	Female	60	48	13,9
23	Female	74	45	12,5
24	Female	73	45	14,4
25	Female	68	48	18,5
26	Male	62	56	15,3
27	Male	68	55	18,4
28	Female	70	43	10,0
29	Female	65	45	17,2
30	Female	66	41	8,5
31	Male	88	48	6,2
32	Female	85	57	8,2
33	Male	83	44	12,9
34	Male	83	54	8,7
35	Male	81	54	8,7
36	Female	83	59	11,3
37	Female	81	51	8,9
38	Female	80	48	6,5
39	Male	82	50	6,0
40	Female	86	52	8,4

41	Male	84	54	7,0
42	Female	87	53	7,7
43	Male	87	52	8,2
44	Male	84	49	6,5
45	Male	83	54	7,0
46	Male	82	49	6,3
47	Male	84	51	8,2
48	Male	83	51	6,5
49	Male	84	50	6,5
50	Male	86	51	7,0
51	Male	81	52	7,8
52	Male	83	56	7,0
53	Male	84	55	7,4
54	Male	80	51	7,5
55	Male	86	48	7,2
56	Male	86	51	7,2
57	Female	85	53	7,7
58	Male	85	54	7,5
59	Male	81	54	7,2
60	Male	83	41	8,9
			**vocabulary**	**fluency**
		**Min:**	39,00	6,00
		**1stQ:**	45,00	7,46
		**Median:**	49,00	9,50
		**Mean:**	48,95	10,80
		**SD:**	4,98	3,88
		**3rdQ:**	53,00	13,89
		**Max:**	59,00	20,00

TRIALS LIST (SCORES Normative AoA)

Word	Trial nº	AoA	Condition	Score Normative
Gato	0	ensayo	ensayo	1.20
Auto	0	ensayo	ensayo	1.50
Mesa	0	ensayo	ensayo	1.30
Perro	0	ensayo	ensayo	1.10
Cama	0	ensayo	ensayo	1.40
Mano	1	temprana	experimental	1.00
Cuchara	2	temprana	experimental	1.80
Caballo	3	temprana	experimental	2.50
Árbol	4	temprana	experimental	2.00
Vaca	5	temprana	experimental	2.20
Casa	6	temprana	experimental	1.60
Puerta	7	temprana	experimental	1.70
León	8	temprana	experimental	2.50
Silla	9	temprana	experimental	1.90
Vaso	10	temprana	experimental	2.10
Gallina	11	temprana	experimental	2.80
Avión	12	temprana	experimental	3.00
Manzana	13	temprana	experimental	2.30
Plátano	14	temprana	experimental	2.40
Lápiz	15	temprana	experimental	3.20
Pantalón	16	temprana	experimental	3.10
Elefante	17	temprana	experimental	3.80
Calcetín	18	temprana	experimental	3.40
Teléfono	19	temprana	experimental	2.60
Flor	20	temprana	experimental	2.70
Globo	21	temprana	experimental	3.30
Conejo	22	temprana	experimental	2.90
Moto	23	temprana	experimental	3.60
Libro	24	temprana	experimental	2.50
Pez	25	temprana	experimental	2.80
Taza	26	temprana	experimental	2.40
Gorro	27	temprana	experimental	3.50
Peineta	28	temprana	experimental	3.20
Estrella	29	temprana	experimental	3.70
Tigre	30	temprana	experimental	3.90
Jirafa	31	temprana	experimental	3.10
Frutilla	32	temprana	experimental	3.80
Yogur	33	temprana	experimental	3.00
Llave	34	temprana	experimental	3.50

Botella	35	temprana	experimental	3.20
Oso	36	temprana	experimental	2.60
Araña	37	temprana	experimental	3.40
Cepillo	38	temprana	experimental	3.60
Rueda	39	temprana	experimental	3.90
Vestido	40	temprana	experimental	3.70
Cuchillo	41	temprana	experimental	3.30
Tambor	42	temprana	experimental	3.50
Limón	43	temprana	experimental	3.10
Tomate	44	temprana	experimental	3.10
Cebra	45	temprana	experimental	2.80
Pincel	46	tardía	experimental	5.10
Semáforo	47	tardía	experimental	5.60
Pulsera	48	tardía	experimental	5.00
Corona	49	tardía	experimental	5.70
Collar	50	tardía	experimental	5.90
Ardilla	51	tardía	experimental	5.50
Lupa	52	tardía	experimental	5.50
Rinoceronte	53	tardía	experimental	5.80
Acordeón	54	tardía	experimental	6.50
Banano	55	tardía	experimental	5.50
Pesebre	56	tardía	experimental	5.20
Maní	57	tardía	experimental	5.80
Ventilador	58	tardía	experimental	5.10
Cadena	59	tardía	experimental	5.30
Carretilla	60	tardía	experimental	5.70
Ampolleta	61	tardía	experimental	5.40
Micrófono	62	tardía	experimental	5.60
Hacha	63	tardía	experimental	6.00
Canasta	64	tardía	experimental	5.60
Pipa	65	tardía	experimental	6.20
Carrusel	66	tardía	experimental	5.90
Raqueta	67	tardía	experimental	5.80
Brocha	68	tardía	experimental	5.50
Remolino	69	tardía	experimental	6.10
Cafetera	70	tardía	experimental	6.80
Candelabro	71	tardía	experimental	7.00
Cenicero	72	tardía	experimental	7.20
Brújula	73	tardía	experimental	6.40
Espuela	74	tardía	experimental	7.50
Espátula	75	tardía	experimental	6.30
Extintor	76	tardía	experimental	6.70
Medidor	77	tardía	experimental	6.90

Urinario	78	tardía	experimental	7.80
Higos	79	tardía	experimental	5.70
Teclado	80	tardía	experimental	5.90
Araucaria	81	tardía	experimental	7.10
Herradura	82	tardía	experimental	7.40
Ancla	83	tardía	experimental	6.50
Grifo	84	tardía	experimental	5.50
Medusa	85	tardía	experimental	6.20
Corcho	86	tardía	experimental	6.80
Interruptor	87	tardía	experimental	6.00
Audífonos	88	tardía	experimental	5.80
Caset	89	tardía	experimental	7.60
Chata	90	tardía	experimental	7.30
Reloj	91	relleno	relleno	4.20
Hoja	92	relleno	relleno	3.00
Guante	93	relleno	relleno	4.00
Basurero	94	relleno	relleno	4.50
Estuche	95	relleno	relleno	4.80
Servilleta	96	relleno	relleno	4.30
Corazón	97	relleno	relleno	3.50
Escalera	98	relleno	relleno	4.10
Helicóptero	99	relleno	relleno	4.70
Falda	100	relleno	relleno	4.40
Durazno	101	relleno	relleno	4.20
Zorro	102	relleno	relleno	4.60
Martillo	103	relleno	relleno	4.90
Caracol	104	relleno	relleno	4.50
Calzón	105	relleno	relleno	5.10
Pingüino	106	relleno	relleno	5.30
Bombilla	107	relleno	relleno	5.40
Copa	108	relleno	relleno	4.00
Sombrero	109	relleno	relleno	3.80
Camisa	110	relleno	relleno	3.60
Lentes	111	relleno	relleno	4.80
Foca	112	relleno	relleno	5.20
Pepino	113	relleno	relleno	4.70
Pulpo	114	relleno	relleno	5.50
Cereza	115	relleno	relleno	4.90
Botón	116	relleno	relleno	4.30
Cebolla	117	relleno	relleno	5.00
Buzo	118	relleno	relleno	5.60
Velador	119	relleno	relleno	5.70
Pito	120	relleno	relleno	5.80
